# Integrated microbiome-metabolome profiling unveils a predictive signature for early recurrence in hepatocellular carcinoma

**DOI:** 10.3389/fmicb.2025.1653249

**Published:** 2025-09-02

**Authors:** Qiqi Chen, Yi Wang, Daoyuan Wu, He Zhang, Qingxin Xia, Dingding Qu

**Affiliations:** Henan Medical Key Laboratory of Tumor Pathology and Artificial Intelligence Diagnosis, Zhengzhou Key Laboratory of Accurate Pathological Diagnosis of Intractable Tumors, Henan Engineering Research Center of Pathological Diagnostic Antibody, Department of Pathology, The Affiliated Cancer Hospital of Zhengzhou University & Henan Cancer Hospital, Zhengzhou, Henan, China

**Keywords:** hepatocellular carcinoma (HCC), intra-tumoral microbiome, early recurrence, diagnostic micro-biomarkers, microbial metabolomics

## Abstract

Hepatocellular carcinoma (HCC) exhibits high recurrence rates post-resection, yet predictive biomarkers remain elusive. Emerging evidence implicates intratumoral microbiota in cancer progression, but its role in HCC recurrence is unexplored. Here, we characterized microbial and metabolic profiles in 90 HCC patients [49 with early recurrence (RFS ≤ 2 years), 41 non-recurrent controls] using 16S rRNA sequencing and LC–MS metabolomics. Recurrent tumors showed reduced microbial diversity (Shannon index, *p* < 0.05) and distinct compositional shifts, including enrichment of Proteobacteria (LEfSe LDA > 4) and depletion of commensals like Akkermansia. A 20-microbial-genus signature predicted recurrence (AUC = 0.81, 95% CI: 0.72–0.91), while a 20-metabolite panel (e.g., resolvin D5, *γ*-glutamylthreonine) achieved superior accuracy (AUC = 0.958, CI: 0.950–0.966). Functional analyses linked recurrence-associated microbiota with disrupted lipid/amino acid metabolism and pro-inflammatory pathways (KEGG, *p* < 0.01). Microbial-metabolite correlation networks revealed strong associations between dysbiotic taxa (e.g., Cyanobacteria) and immunomodulatory metabolites (*r > 0.6, *p* < 0.05). This study identifies intra-tumoral microbiome-metabolome signatures as novel biomarkers for HCC recurrence, offering mechanistic insights into microbial regulation of the tumor microenvironment and clinical tools for post-surgical risk stratification.

## 1 Introduction

As the predominant form of primary liver malignancy, hepatocellular carcinoma (HCC) represents a primary cause of global cancer mortality, accounting for the vast majority of hepatic neoplasms with invasive potential. Clinically, hepatocellular carcinoma manifests with subtle early symptoms, rapid advancement, and frequent recurrence. Delayed diagnosis and therapeutic resistance contribute to its unfavorable outcomes. Despite improvements in clinical trials and diagnoses, HCC still has a high mortality rate, due to 70% recurrence and lung metastasis after surgical resection.

Surgical treatment, including hepatic resection and liver transplantation, is a potentially curative therapeutic option for HCC. Indications for partial resection include unilobar tumors lacking vascular involvement and liver metastases without cirrhosis. The Child-Pugh score guides patient selection for resection by assessing hepatic function through biochemical markers (bilirubin, albumin), coagulation status (prothrombin time), and the detection of ascites or encephalopathy. However, the 5-year recurrence rate following liver resection for HCC approaches 75% (Sugawara and Hibi, 2021; Rajendran et al., 2022), representing a persistent clinical dilemma that extends to patients presenting with single tumors ≤2 cm in diameter (Roayaie et al., 2013).

Numerous studies claim that intratumoral bacteria reside not only within malignant cells but also in immune cells, with their microbial profiles exhibiting tumor-specific variations (Nejman et al., 2020). Polymorphic microbiomes are a new hallmarks of cancer (Hanahan, 2022). Due to the physiological and anatomical bidirectional association between the liver and the intestine, recent studies mostly explore the important contribution of gut microbiota in hepatic inflammation, fibrosis and tumor development through the “gut-liver axis” (Bourzac, 2014; Tilg et al., 2022; Hov and Karlsen, 2023), while the characteristics and metabolic differences of intra-tumoral microbiota of HCC patients were rarely reported. In our initial investigation, preliminary characterization of PLC-associated microbiota across histopathological variants and clinical prognoses uncovered microbiome disparities that distinguished: ① tumor from adjacent non-tumor tissue, ② different histological subtypes, and ③ patients with divergent clinical outcomes (Qu et al., 2022). However, unlike the well-established causal effect of *H. pylori* on the progression of gastric cancer, no specific intra-tumoral bacterial community (genus) has been identified to cause HCC recurrence. The precise role of intratumoral microbiome components in modulating HCC progression, whether beneficial or detrimental, has yet to be fully elucidated.

Hence, based on previous findings, this study intends to expand the cohort and conduct multi-omics sequencing to map the characteristics of intra-tumoral microbiota and metabolic differences in HCC patients, and to develop a microbiome-derived predictive classifier for postoperative HCC recurrence. This study may enable us to reveal the complex relationship between intra-tumoral microbiota dysbiosis and HCC recurrence and provide a novel microbiome-based intervention strategy to enhance survival outcomes in HCC management.

## 2 Materials and methods

### 2.1 Participants information and FFPE samples collection

The study commenced with an initial discovery cohort comprising HCC patients without recurrence after surgery (>2 years RFS, *n* = 41), contrasted with stage-comparable HCC cases with PFS < 2 years (*n* = 49) at the Affiliated Tumor Hospital of Zhengzhou University (Henan Province, China) from 2017 to 2018. Table 1 displays the patients in detail along with their pathological and clinical data. Postoperative staging of specimens was conducted by a specialized pathologist, following the TNM staging system and the stage groupings (I-IV) established by the American Joint Committee on Cancer. All clinical covariates in Table 1 were included as adjustment variables in multivariate Cox models. FFPE specimens were procured following our established protocol (Qu et al., 2022). For each specimen, the initial sections from the FFPE blocks were excluded, followed by the collection of 2 mm tumor tissue cores using a sterile drill. These samples were then transferred into pre-sterilized 2 mL microcentrifuge tubes. The research protocol obtained formal ethical approval from the Institutional Review Board of the Affiliated Cancer Hospital of Zhengzhou University and conformed to the principles of the Declaration of Helsinki and Good Clinical Practice guidelines. Prior to enrollment, all participants provided written informed consent after a detailed explanation of the study. Additionally, they authorized the use of their anonymized data for future research.

**Table 1:** Clinicopathological characteristics of all participants.

Clinical and pathological indexes	Recurrence (*n* = 49)	Non-recurrence (*n* = 41)	*P*-value
Age, years, median (range)	56.3 (33–74)	50.7 (30–70)	0.501
Gender (male/female)	42/7	33/8	0.508
Surgery date	Feb2017-Nov2018	Jan2017-Dec2018	
Hepatitis B, number	48	38	0.327
Liver cirrhosis, number	34	32	0.355
Capsular invasion, number	34	15	0.002
Microvascular invasion, number	24	5	<0.001
Differentiation			
Moderately	37	34	0.391
Poorly	12	7	
AJCC stage			
IA	3	1	0.001
IB	24	35	
ssswII	22	5	
Nerve invasion, number	/	/	
Lymphatic metastasis, number	/	/	

*p*-values were calculated by Fisher’s exact test.

### 2.2 DNA extraction and bacterial 16S rRNA sequencing

Total microbial DNA was isolated from FFPE tissues using the QIAamp DNA FFPE tissue kit (QIAGEN, CA, United States), with all samples were immediately frozen and stored at −20°C prior to processing. The DNA concentration and purity were assessed spectrophotometrically (NanoDrop 2000, Thermo Fisher Scientific, Wilmington, United States) according to the manufacturer’s instructions, where samples with A260/280 ratios of 1.8–2.0 were retained for downstream analysis.

DNA samples were amplified, libraries were constructed, and sequencing was performed on an Illumina MiSeq platform by Shanghai Majorbio Bio-Pharm Technology Co., Ltd., China. The 16S rDNA hypervariable V4 region was amplified using barcoded primers (341F: 5′-CCTAYGGGRBGCASCAG-3′; 806R: 5′-GGACTACNNG GGTATCTAAT-3′) incorporating Illumina adapter sequences. PCR products were purified and normalized before pooled library preparation. The amplification primers incorporated MiSeq sequencing adapters and unique single-index barcodes, enabling direct pooling and sequencing of PCR amplicons (Caporaso et al., 2012), with a minimum sequencing depth of 10,000 reads per sample. The 16S rRNA gene (variable region 4 [v4]) pipeline data incorporated phylogenetic and alignment methods to enhance taxonomic resolution. Following demultiplexing using sample-specific barcodes introduced during PCR amplification, paired-end reads were assembled with USEARCH (v7.0.1090) (Edgar, 2010).

### 2.3 Microbiome profiling analysis workflow

Paired-end V4 reads were assembled into contigs using FLASH (v1.2.11), followed by quality filtration (error rate < 0.5%; length ≥ 200 bp) through Trimmomatic and QIIME (Bolger et al., 2014). PhiX control sequences were identified by BLASTN alignment (E-value ≤ 1e-5). Primer sequences were trimmed, followed by chimera detection using UCLUST *de novo* mode (v11.0.667), and screened for human-associated contaminants using Bowtie 2 (Langmead and Salzberg, 2012). Chloroplast/mitochondrial contaminants excluded using an RDP classifier (confidence threshold 50%). Taxonomic assignment was performed using Resphera Insight (v1.0) against SILVA Database v138 at 99% OTU similarity (Daquigan et al., 2017; Drewes et al., 2017). Diversity analyses (*α*/*β*) and principal coordinates analysis were conducted in QIIME and R. Differential abundance assessed via nonparametric statistical methods. Differential abundance analysis of taxonomic abundance was performed using the negative binomial test (DESeq: FDR-adjusted *p* < 0.05) (Anders and Huber, 2010). Non-metric multidimensional scaling (NMDS) and principal coordinate analysis (PCoA) were performed. Nonparametric Kruskal-Wallis rank-sum test and Wilcoxon matched-pairs signed rank test were used to perform linear discriminant analysis (LDA) effect size (LEfSe) analysis (LDA score >3.0, Kruskal-Wallis *p* < 0.01) to detect discriminative taxa with significant differences between HCC recurrence and Non-recurrence cases (Lozupone et al., 2011).

### 2.4 Metabolite extraction and LC–MS untargeted metabolomics and analysis

Tissues samples were homogenized in liquid nitrogen and extracted using prechilled 80% methanol and 0.1% formic acid. After vortexing, the samples were kept on ice for 5 min and centrifuged at 15,000 rpm (4°C) for 5 min. The supernatant was diluted to 53% methanol by LC–MS-grade water, followed by another centrifugation step at 13,000 × g (4°C) for 15 min. Finally, the clarified supernatant was then collected to sample vials for LC–MS/MS analysis.

An UHPLC-Q Exactive HF-X system (Thermo Fisher Scientific) coupled with an electrospray ionization (ESI) source was employed for untargeted metabolomics profiling, operating in both positive and negative ion modes. Data were acquired in data-dependent acquisition (DDA) mode, covering a mass range of 70–1,050 m/z. Principal component analysis (PCA) was conducted using the ropls R package (Version 1.6.2), and used 7-cycle cross-validation to access model robustness. Significantly altered metabolites were identified based on variable importance in projection (VIP > 1) from OPLS-DA and statistical significance (*p* < 0.05, Student’s *t*-test). Pathway enrichment analysis was performed using KEGG database (KEGG),^1^ and significantly perturbed metabolic pathways were determined via Fisher’s exact test (Scipy.stats, Python packages).^2^

### 2.5 Statistical analysis

Statistical analyses were conducted using SPSS 27.0 (SPSS Inc., United States) and GraphPad Prism 8.0. Multivariate Cox proportional hazards regression was applied to adjust for confounding factors such as capsular and microvascular invasion. Group comparisons for continuous variables were performed using the Student’s *t*-test (normally distributed data) or the Wilcoxon rank-sum test (non-parametric data), while categorical variables were assessed via Fisher’s exact test. A two-sided *p-value <0.05 was considered statistically significant. Differences in alpha-diversity indices and metabolite concentrations between groups were evaluated using the Student’s *t*-test. Microbiome–metabolome associations were examined using Spearman’s correlation and displayed using the R software (version 3.6.1).

## 3 Results

### 3.1 Elevated tumor microbial diversity correlates with improved prognosis in resected HCC patients

**Figure 1:**
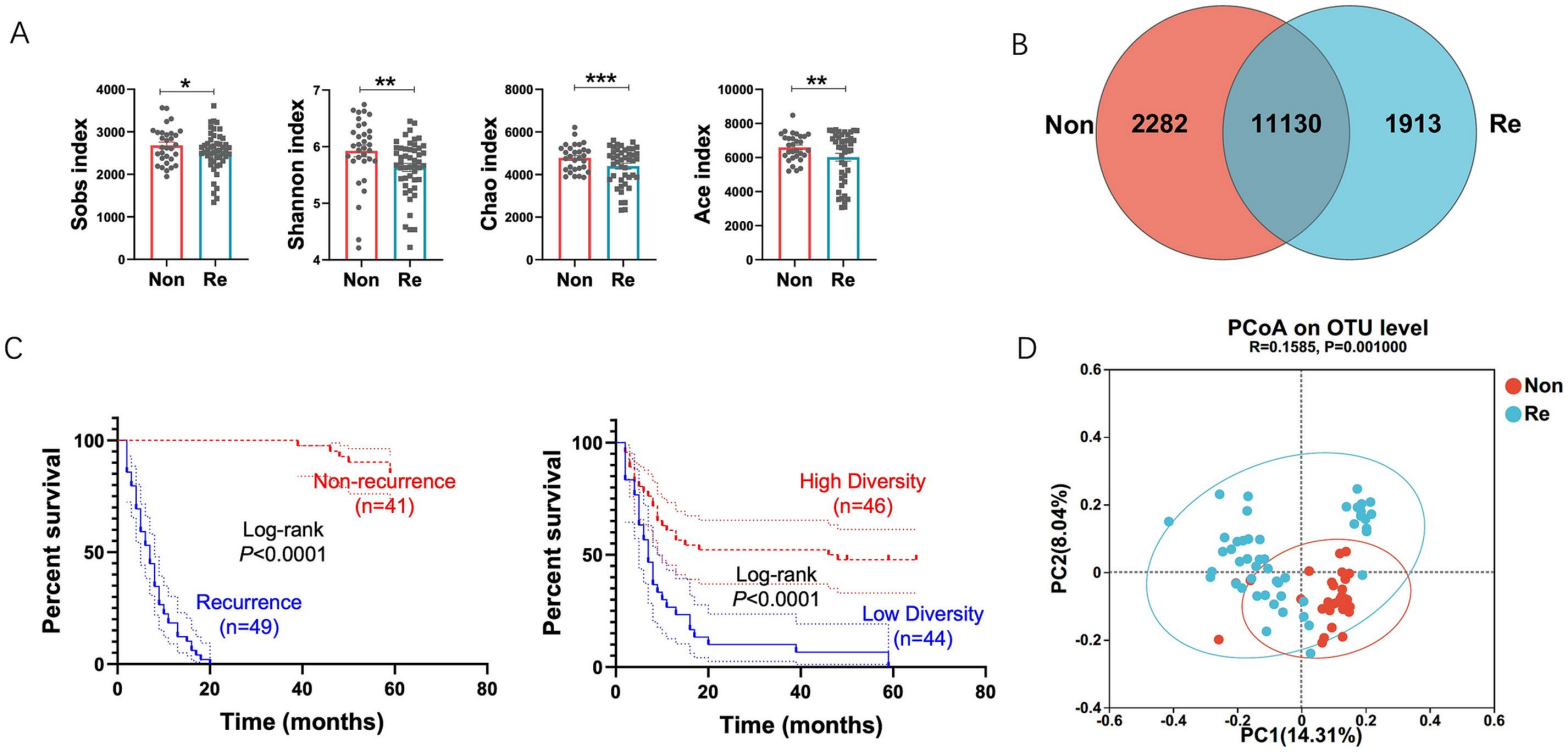
Intra-tumoral microbial diversity between HCC recurrence and non-recurrence patients. **(A)** *Alpha* diversity estimated by the Sobs index, Shannon index, Chao index and ACE index in samples of each group of patients. **(B)** Venn diagram displaying the degree of overlap of bacterial OTUs between the HCC recurrence and non-recurrence groups. **(C)** Kaplan–Meier plot of cohort HCC recurrence patients defined by alpha diversity. **(D)** Principal coordinates analysis (PCoA) of bacterial beta diversity based on the unweighted UniFrac distances. Values were expressed as mean ± SEM (*p < 0.05, paired *t*-test).

To elucidate the role of intra-tumoral microbiota in HCC prognosis, we analyzed a discovery cohort comprising stage-matched patients who underwent curative resection. Participants were stratified into two groups: (1) non-recurrence cases (RFS ≥ 2 years) and (2) early recurrence cases (RFS ≤ 2 years). The cohorts were well-matched in terms of age, sex, Barcelona Clinic Liver Cancer (BCLC) stage, and treatment history (Table 1; Figure 1C). We found that microbial diversity, as estimated by the Shannon, Ace, Sobs, and Chao indices, was significantly decreased in the recurrence groups (Figure 1A). A Venn diagram of the composition of the microbiota in the tumor tissues showed that tumor tissues from HCC recurrence patients had lower microbial diversity (Figure 1B).

To investigate the prognostic value of intra-tumoral microbial diversity in HCC, we stratified patients into high- and low-diversity groups based on median Shannon index values. Univariate Cox regression analysis indicated that the high-diversity group had a substantially longer recurrence-free survival (RFS) (median: 47 months) compared to the low-diversity group (median: 7 months; Figure 1C), underscoring the clinical relevance of microbial alpha diversity. To further characterize microbiome-host interactions, we examined phylogenetic patterns distinguishing recurrence-associated microbial communities. Beta-diversity analysis via principal coordinate analysis (PCoA) based on unweighted UniFrac distances demonstrated: (1) distinct clustering patterns between recurrence and non-recurrence groups, and (2) greater phylogenetic homogeneity within each group (Figure 1D).

### 3.2 Phylogenetic profiling of tumor-resident microbiota in HCC patients

**Figure 2:**
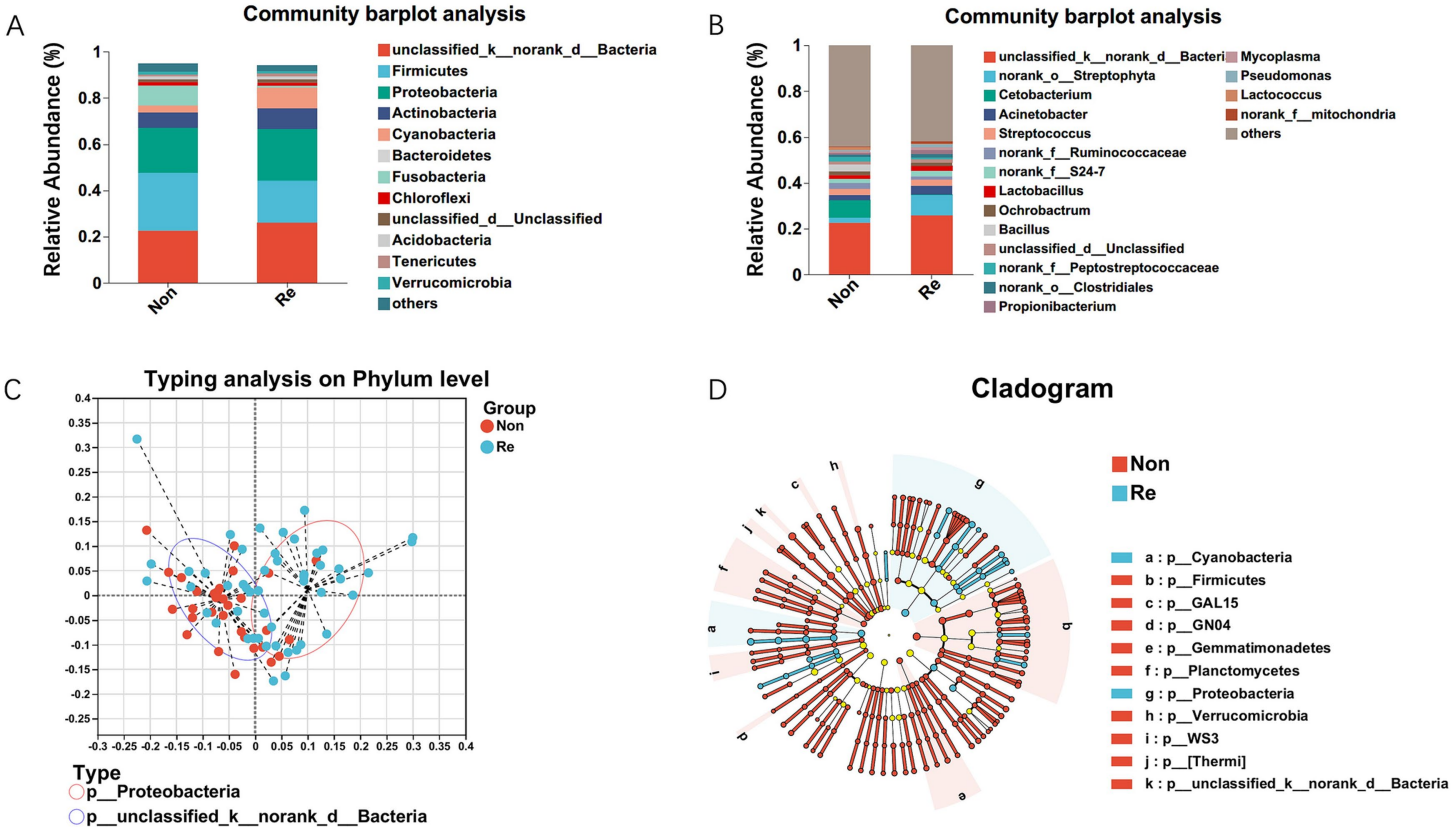
Intra-tumoral microbial profile differs in HCC recurrence and non-recurrence patients. Composition of microbiota at the phylum level **(A)** and genus level **(B)** between the two groups, respectively. **(C)** Enterotype analysis at the phylum level between HCC recurrence and non-recurrence groups. The data are most naturally separated into two clusters, as determined by the Calinski-Harabasz (CH) index and represented using principal coordinate analysis (PCoA). The shapes and colors of the points indicate samples from each individual from various months. The colored ellipses indicate the 0.95 confidence interval (CI) ranges within each enterotype group. **(D)** LEfSe analysis used to display the marked differences in the predominance of bacterial communities between HCC Non-recurrence and recurrence groups. Only taxa with an LDA value > 4 are presented. Circles indicate phylogenetic levels; diameter and color of each circle represent its abundance and enterotype, respectively.

Building upon the established association between intratumoral microbiome diversity and postoperative outcomes, we conducted phylogenetic characterization to identify recurrence-specific microbial signatures in HCC. Initial comparative analysis revealed conserved microbial architectures across recurrence/non-recurrence cohorts. We first identified operational taxonomic units (OTUs) with median relative abundance >0.01%, then conducted differential abundance analysis using Wilcoxon rank-sum with Benjamini-Hochberg false discovery rate (FDR) adjustment (Figure 2A). Dominant bacterial genera maintained consistent distribution patterns between groups, as evidenced by genus-level compositional profiling is shown in Figure 2B. Enterotype analysis was performed to assess the community composition of the dominant phyla within HCC recurrence patients using a clustering approach. These results suggested that the HCC recurrence group was mainly clustered in Proteobacteria (Figure 2C). In addition, high-dimensional comparative analysis using LEfSe (Linear Discriminant Analysis Effect Size) revealed significant disparities in bacterial community predominance between HCC non-recurrence and recurrence cohorts (Figure 2D). The HCC recurrence cases exhibited a predominance of Cyanobacteria and Proteobacteria at the phylum level and marked decreases in the other commensal microbiota.

### 3.3 HCC recurrence patients exhibit a distinct intra-tumoral microbial communities

**Figure 3:**
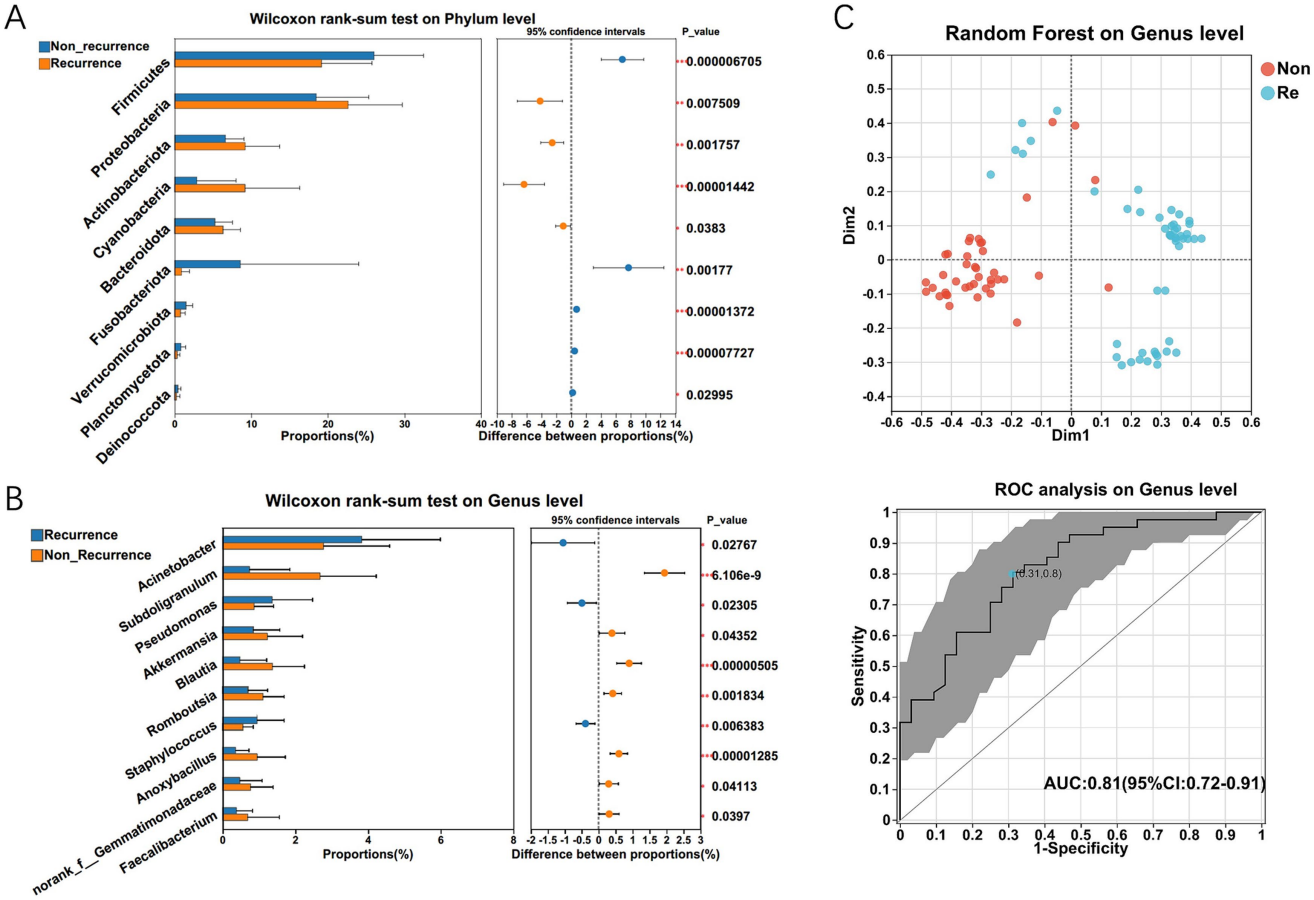
The significant microbial difference and the identification of the differential bacterial taxa associated with HCC recurrence. The differentially expressed bacterium between HCC recurrence and non-recurrence patients on phylum **(A)** and genus level **(B)** respectively. Multiple testing correction using two-tailed Wilcoxon test and FDR; *p < 0.05; CI calculated by the bootstrap method using 95% CI. **(C)** Twenty genera enriched in recurrence cases were selected as the optimal marker set by random forest models, and The POD index achieved an AUC value of 0.81 with 95% CI of 0.72–0.91 between HCC recurrence and non-recurrence groups.

Considering the significant difference in predominant bacterial communities between the HCC Non-recurrence and recurrence groups by LEfSe analysis, we further characterized the differentially abundant taxa at both phylum and genus levels. At the phylum level, the Wilcoxon rank-sum test revealed significant differences in bacterial communities between the two groups, including Firmicutes, Proteobacteria, Cyanobacteria, Chloroflexi, Verrucomicrobia, and Gemmatimonadetes (Figure 3A). The genus levels of the bacterial communities differentially expressed between the two groups are shown in Figure 3B. To identify the differential bacterial taxa associated with HCC recurrence, we constructed a random forest classifier model that could specifically distinguish HCC recurrence samples from non-recurrence samples (Figure 3C). To evaluate the predictive performance of recurrence-associated microbial signatures, we implemented a 10-fold cross-validated random forest model. Twenty genera demonstrating elevated abundance in recurrence cases were subsequently subjected to receiver operator characteristic (ROC) curve analysis, with the probability of disease (POD) index serving as the classification metric. The POD index demonstrated strong discriminative capacity (AUC = 0.81; 95% CI: 0.72–0.91) for identifying post-operative recurrence (Figure 3C). This microbial signature-based approach demonstrated clinically relevant predictive potential for HCC recurrence.

### 3.4 Potential biological functions of bacterial communities within HCC recurrence patients

**Figure 4:**
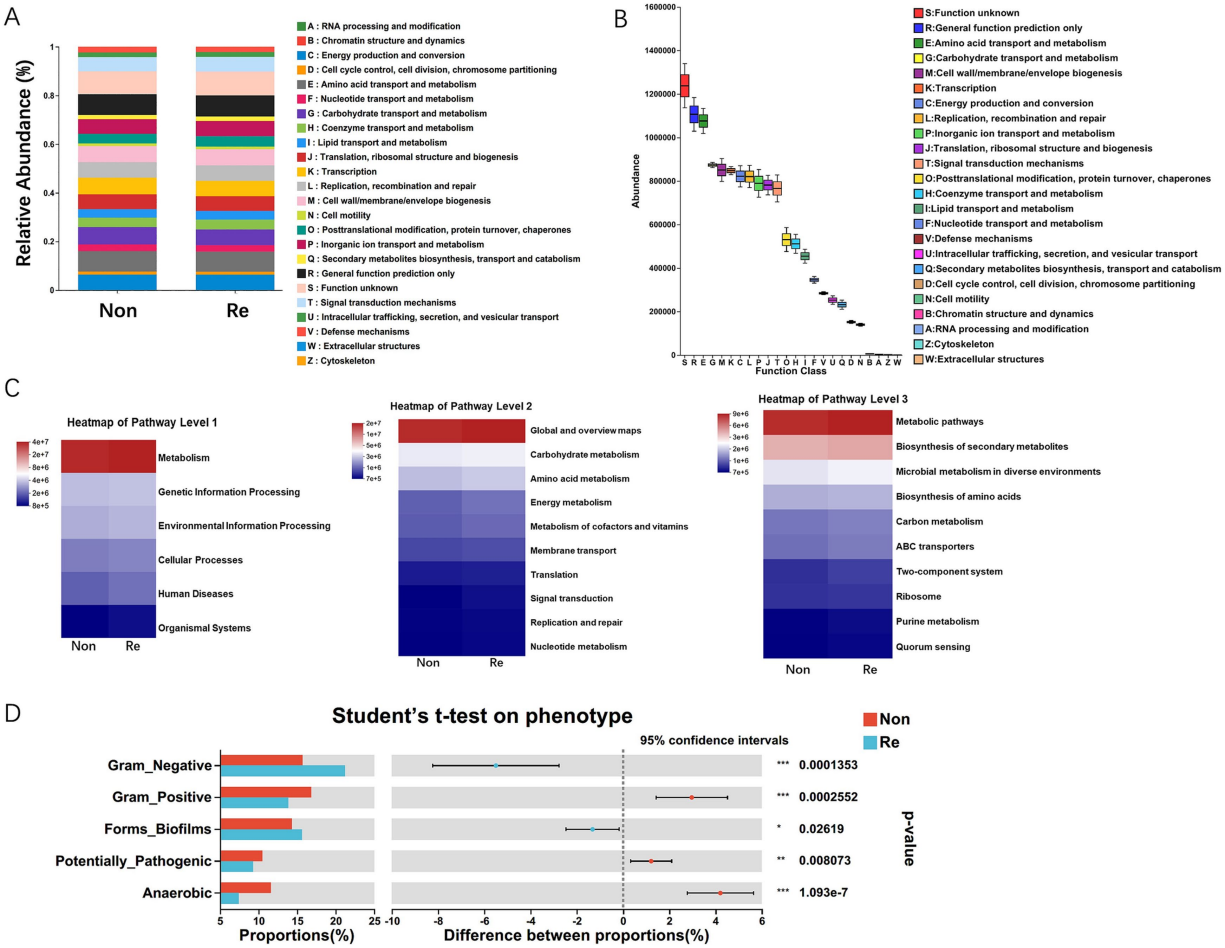
The functional prediction analysis of intra-tumoral microbial communities. **(A)** The difference of relative abundance of PICRUSt inferred function between HCC recurrence and non-recurrence patients. **(B)** PICRUST1 combined with the EggNOG database to predict the function of intra-tumoral microbiota in HCC patients. **(C)** PICRUSt2 combined with the KEGG database to predict the function of bacterial microbiota in tissues, showing the results of the KEGG pathway in Level 1, 2, and 3. **(D)** Microbial phenotypic functional prediction based on BugBase database (*p < 0.05; **p < 0.01; ***p < 0.001).

To predict potential metabolic functions of HCC intra-tumoral microbiota, we performed PICRUSt2 analysis, which phylogenetically infers uncharacterized community functions. And then integrated 16S rRNA amplicon sequencing data with both EggNOG orthology and KEGG pathway annotations (Figure 4A). The functions of bacterial communities within HCC recurrence patients were predicted to be mainly involved in metabolism, among which amino acid- and carbohydrate-related transport/metabolic pathways demonstrated significantly higher abundance compared to other functional categories (*p* < 0.01). Similarly, the 16S rRNA sequencing data combined with KEGG functional predictions revealed that bacterial functions were primarily related to carbohydrate and amino acid metabolism pathways (Figures 4B,C). Furthermore, KEGG level-3 functional profiling revealed HCC microbiome’s enriched involvement in secondary metabolite production and adaptive metabolic pathways across ecological niches (Figure 4C). Via the BugBase potential prediction of phenotypic functions of intra-tumoral microbiota, several potential microbial phenotypes were found to be different in HCC recurrence patients, encompassing: (i) Gram-negative/positive bacteria, (ii) biofilm-producing strains, (iii) virulence factor-containing species, and (iv) anaerobic microorganisms (Figure 4D).

### 3.5 The microbiome-associated metabolic profiles in HCC recurrence microenvironments

**Figure 5:**
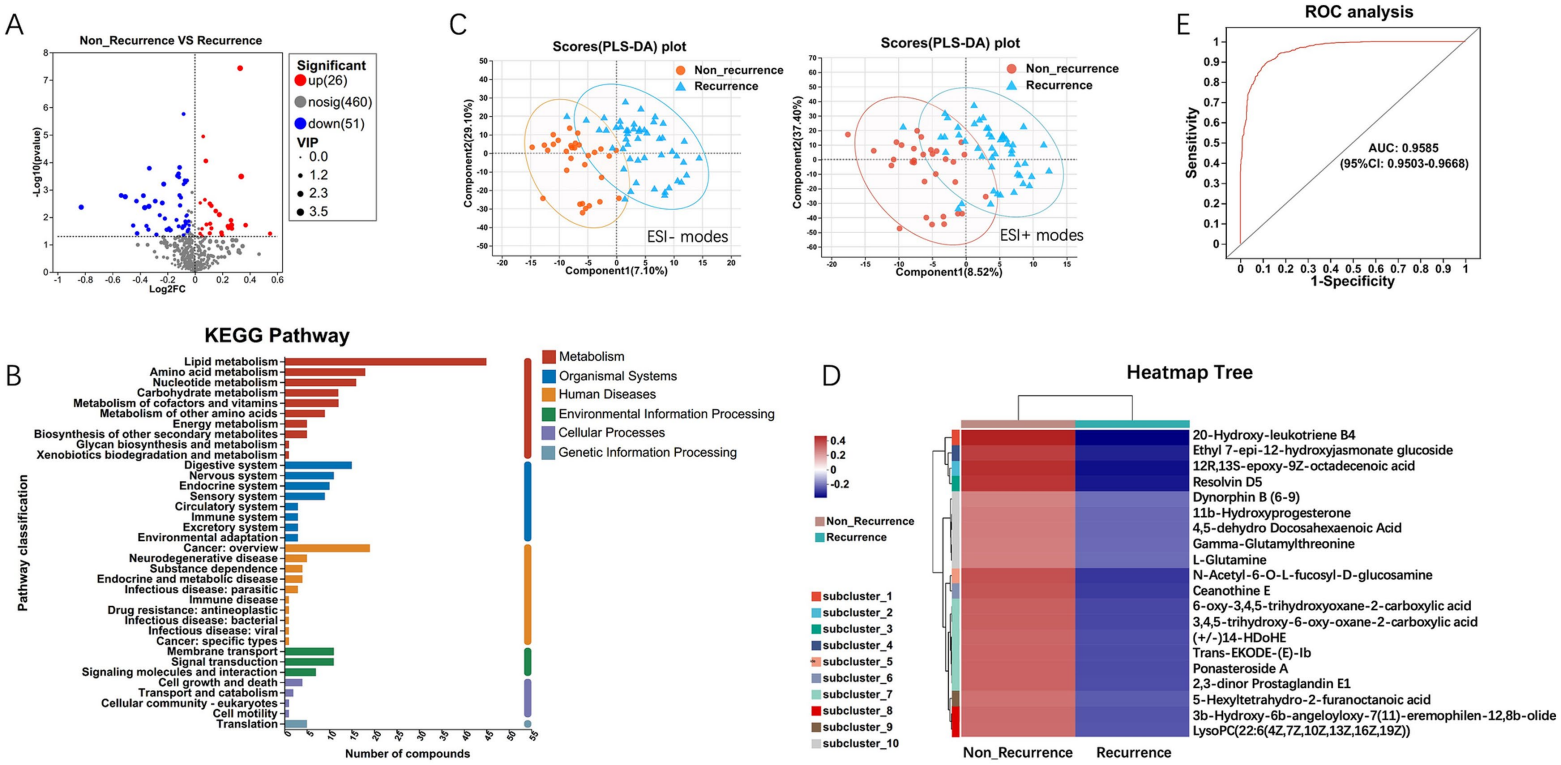
Differential metabolites analysis between HCC recurrence and non-recurrence patients. **(A)** Volcano plot of differential metabolites. **(B)** KEGG pathway enrichment analysis displays the significantly enriched items of metabolites. **(C)** PLS-DA (Partial Least Squares Discriminant Analysis) of metabolomics data. Each sample was represented as one spot in the score plots, and the HCC non-recurrence and recurrence groups were separated in ESI- (left) and ESI + (right) modes. **(D)** Expression abundance of differential metabolites. The colors from blue to red indicate the metabolite expression abundance from low to high. **(E)** The 20 differential metabolites with higher abundance were selected as the optimal marker set by random forest models, and The POD index achieved an AUC value of 0.9585 with 95% CI of 0.9503–0.9668 between HCC recurrence and non-recurrence groups.

To extend understand the role of microbiota in HCC recurrence, we applied an untargeted metabolomics approach to explore the ability of microbial metabolites to regulate the tumor microenvironment. Univariate statistical analysis showed that a total of 77 metabolites were significantly different (*p* < 0.05, fold change > 2) in HCC recurrence patients, with 51 downregulated and 26 upregulated metabolites (Figure 5A). KEGG pathway enrichment analysis suggested that the significantly enriched metabolites were metabolism-related pathways, including lipid, amino acid, nucleotide, and carbohydrate metabolisms (Figure 5B). The Multivariate analysis via PLS-DA (Partial Least Squares Discriminant Analysis) revealed clear clustering segregation, indicating the existence of different biological characteristics between the two groups (Figure 5C). Each sample was represented as one spot in the score plots, and the HCC non-recurrence and recurrence groups were separated in ESI− and ESI + modes.

**Figure 6:**
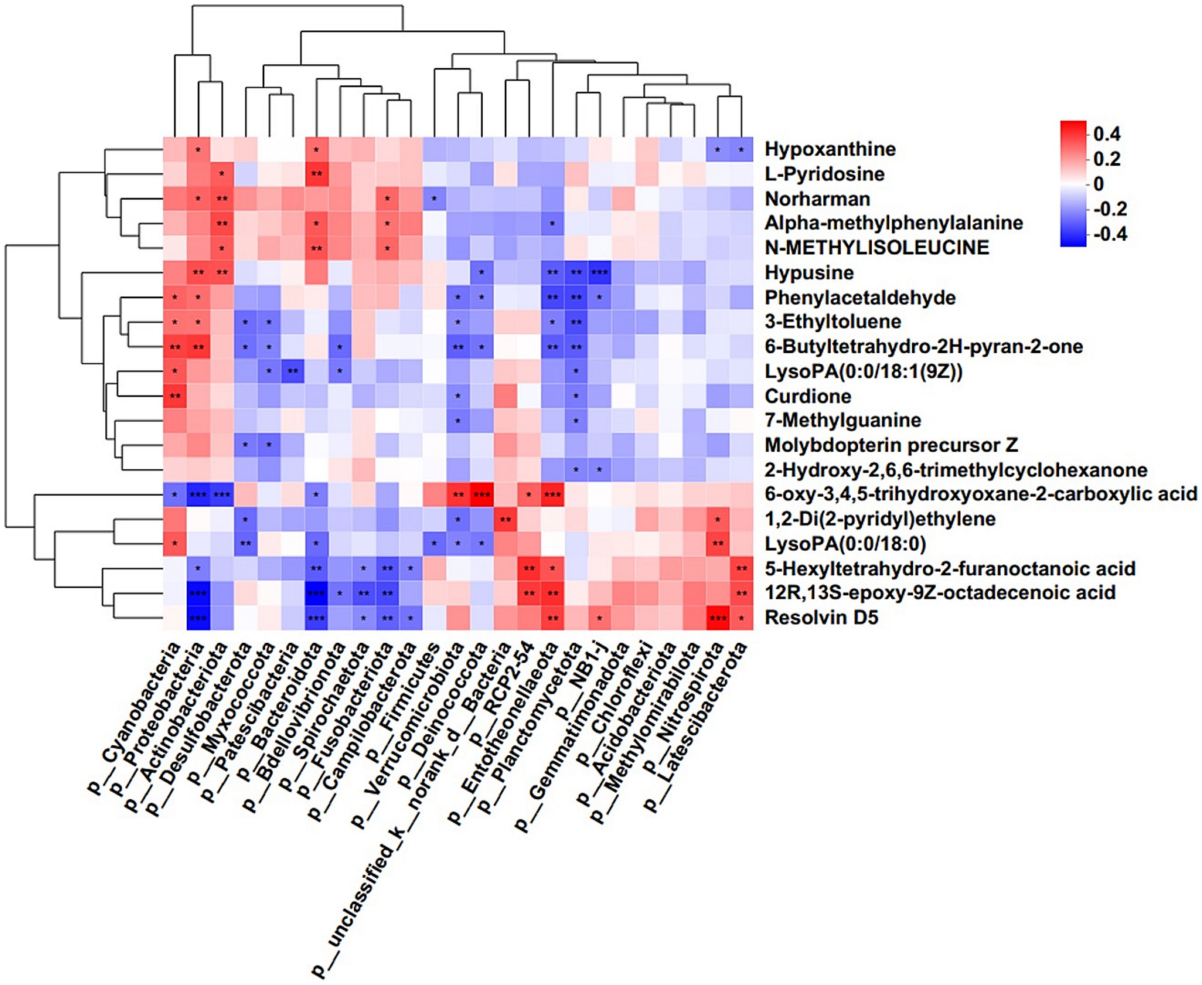
Correlation analysis of microbes and metabolites. Each lattice represents a coefficient by Pearson’s correlation analysis, each column represents a phylum, each row represents a metabolite. Red represents a positive correlation and blue represents a negative correlation (*p < 0.05; **p < 0.01).

Further, hierarchical clustering analysis (HCA) was subsequently employed to compare metabolite abundance patterns between the two groups. The results showed that the metabolites, including 20-Hydroxy-leukotriene B4, Resolvin D5, Gamma-Glutamylthreonine, L-Glutamine, Ceanothine E, Ponasteroside A showed a downregulation trend (Figure 5D). Guided by HCA clustering patterns, we established a diagnostic metabolite signature comprising the top 20 differentially abundant metabolites (VIP > 1.5, FDR < 0.01). ROC curve analysis showed the probability of disease (POD) index had excellent exceptional discriminative capacity (AUC = 0.958, 95%CI: 0.950–0.966) for distinguishing post-operative recurrence (Figure 5E). Mechanistically, Spearman rank correlation (FDR < 0.05) identified robust associations between recurrence-enriched microbes (LDA > 3) and dysregulated metabolites (*p* < 0.01), suggesting microbial modulation of tumor metabolic pathways. As shown in Figure 6, the top 20 differential metabolites strongly correlated with the top 25 altered microbial phyla, indicating that the aberrantly enriched metabolites in HCC recurrence cases may result from dysbiosis of tumor microflora or their interactions. To better illustrate these relationships, we generated a heatmap (Supplementary Figure S1) displaying the Spearman correlation coefficients, revealing statistically significant positive and negative associations (*p* < 0.05) between specific metabolites and distinct microbial phyla. These findings suggest that intra-tumoral microbiota may influence HCC recurrence through metabolic reprogramming, providing potential insights into the microbial-metabolite interplay in tumor progression.

## 4 Discussion

HCC remains a leading cause of cancer-related mortality and requires extensive effort for early detection to mitigate its adverse effects. Despite the numerous liver resections reported in the studies, very little data are available for effectively preventing HCC recurrence. Despite decades of translational research, clinically actionable biomarkers for hepatocellular carcinoma (HCC) remain limited. Since the 1960s, serum alpha-fetoprotein (AFP) quantification combined with radiographic imaging has constituted the diagnostic cornerstone, with this paradigm remaining essentially unchanged for over six decades; however, AFP’s role as a “gold standard” predictor, the role of AFP continues to be highly controversial because of its limited specificity and the ambiguous biology behind the connection between AFP and HCC. The treatment and prevention of HCC remains challenging and is largely predicated on early diagnosis and recurrence. Therefore, the persistent limitations of current recurrence prediction paradigms underscore the critical need for novel biomarker discovery and innovative prognostic strategies in HCC management.

A novel potential cancer hallmark based on intra-tumoral microbiota is emerging. Owing to the physiological and anatomical bidirectional association between the liver and intestine, gut microbiota potentially influences hepatic pathologies, particularly hepatocellular carcinoma, through vascular and portal venous translocation of microbial components and metabolic products (Albillos et al., 2020; Pabst et al., 2023). Accumulating evidence has elucidated the mechanistic contributions of the gut-liver axis to hepatic pathophysiology, particularly in driving inflammatory cascades, fibrogenic progression, and hepatocarcinogenesis. Other studies have reported the connections between digestive tract diseases and oral (Irfan et al., 2020; Herremans et al., 2022) or gut microbiome (Sung et al., 2020; Liu et al., 2022) with taxonomic resolution based on 16S rRNA gene sequencing, including early HCC with cirrhosis (Ren et al., 2019; Zhang et al., 2021). Importantly, the gut microbiota has also been identified as a promising noninvasive biomarker for the early HCC detection, highlighting its clinical utility in disease classification (Ren et al., 2019; Huang et al., 2020). However, the compositional dynamics and functional implications of tumor-resident microbiota during HCC malignant progression remain poorly characterized. Emerging studies indicate that the intra-tumoral microbiome is a component of the tumor microenvironment and has become one of the new hallmarks of cancer, which can induce inflammation and immune response to affect tumorigenesis and development (Cullin et al., 2021; Matson et al., 2021; Sepich-Poore et al., 2021; Hanahan, 2022). Since intra-tumoral microbiota are supposed to be closer to the tumor tissues, shaping a microenvironment that may be relevant to the pathological process of HCC, it is necessary to determine whether the metataxonomic characteristics and metabolic differences of intra-tumoral microbiota can be used as a new potential biomarker for HCC recurrence prediction. Our initial investigations delineated microbiome signatures across histopathological subtypes of primary liver cancer (PLC), revealing bacterial taxa whose abundance correlated with clinical prognosis. The intra-tumoral microbial communities and compositional differences associated with HCC progression and early recurrence were emphasized and highlighted in this study.

Intra-tumoral microbiota dysbiosis may contribute to HCC progression. Our previous study has characterized for the first time the intra-tumoral microbial community profiling of FFPE samples from PLC patients with different prognoses and different histopathological subtypes using 16S rRNA MiSeq sequencing, revealing a significant microbial population difference in PLC patients. Recent studies have shown that the intra-tumoral microbiota may contribute to the promotion of the initiation and progression of cancers by DNA mutations, activating carcinogenic pathways, promoting chronic inflammation, the complement system, and initiating metastasis, and regulating cancer cell physiology and the immune response through different signaling pathways, including ROS, *β*-catenin, TLR, ERK, NF-κB, and STING, among others (Fu et al., 2023; Xue et al., 2023; Yang et al., 2023). Similar to the gut microbiota regulating host immune responses, the intra-tumoral microbiota can also shape the local immune responses of the tumor microenvironment, which further affects tumor progression by either enhancing or decreasing antitumor immune responses and inducing different immunotherapy efficacies and outcomes (Nejman et al., 2020; Siegel et al., 2020). Combined with the previous results on the intra-tumoral microbiome of PLC patients, this study aimed to enroll HCC patients without lymph node metastasis and capsule invasion, who were grouped by relapse after liver surgical resection in 2 years; and conduct multi-omics sequencing to comprehensively characterize the microbial landscape and their metabolic differences in patients with early HCC recurrence. Clinically, cohort studies have revealed that patients with HCC recurrence exhibit distinct intra-tumoral microbial communities and composition and lower microbial diversity. Consistent with its known immunometabolic roles- promoting anti-tumor CD8^+^ T-cell infiltration via butyrate production (Bae et al., 2022; Cani et al., 2022), *Akkermansia* abundance was markedly reduced in HCC recurrence group. Although beyond this biomarker discovery study, future work should integrate spatial transcriptomics/multiplex IHC to map microbial niches, metabolite gradients, and immune infiltrates (e.g., PD-1^+^ TILs, IL-17 levels) in resection specimens-this may elucidate how microbial metabolites locally modulate anti-tumor immunity. For clinical prediction of early HCC recurrence, we established a 20-genera classifier via random forest model with remarkable classification accuracy in predicting early HCC recurrence. As expected, unique intra-tumoral microbial communities and composition caused significant metabolic differences, also exhibiting a powerful classification with high POD indices. These results indicate that distinct intra-tumoral microbiota-targeted biomarkers may be potential predictive tools for early HCC recurrence.

To our knowledge, this is the first cohort study focusing on the intra-tumoral microbial community characteristics and their metabolic differences in HCC recurrence patients with adjustments for clinical confounders and attempts to filter the crucial bacterial candidates and their differential metabolites that may contribute to HCC development. While this study provides novel insights into the intra-tumoral microbiome-metabolome axis in HCC recurrence using 16S rRNA sequencing, it has limitations. The taxonomic resolution of 16S data restricts detailed characterization of microbial functions and non-bacterial components (e.g., viruses, archaea). Future studies utilizing shotgun metagenomic sequencing on fresh-frozen tissues are warranted to validate our biomarker signatures at the strain level, investigate the functional potential of the tumor microbiome, and explore the roles of viral/phage and archaeal communities in recurrence mechanisms. Integrating microbial biomarkers with conventional diagnostic modalities could enhance postoperative HCC management, enabling precise recurrence prediction through minimally invasive analysis of both fresh and FFPE specimens. In addition, while our study identified distinct intra-tumoral microbial signatures in HCC recurrence, we recognize that clinical-pathological factors such as capsular invasion and microvascular invasion, which are more prevalent in the recurrence group, may independently influence outcomes. Although these variables were statistically adjusted in our multivariate analysis, their confounding effects cannot be entirely ruled out. Future studies with stricter cohort matching or stratification by these factors are warranted to validate the microbial biomarkers independently. Unavoidably, the cross-sectional nature of the present study prevented us from elucidating the mechanisms and longitudinal view of relevance; in future studies, a validation cohort and independent diagnosis cohorts need to be carried out to further evaluate the potential of the intra-tumoral microbiome as a novel prognosis-predictive value of the HCC recurrence risk signature. Although our data reveal significant correlations between microbial taxa/metabolites and HCC recurrence, causality must be established via functional models. Future studies using germ-free mice colonized with recurrence-associated microbiota, fecal microbiota transplantation (FMT) in HCC models, and *in vitro* metabolite assays are planned.

## Data Availability

The microbiome (16S rRNA sequencing) and metabolome (LC–MS) raw data have been deposited into the Mendeley Data repository (V2) with the dataset identifier DOI: 10.17632/4gbjp9sn98.1. Any additional information required to reanalyze the data reported in this paper is available from the lead contact upon request.

## Footnotes

^1^ http://www.Genome.jp/kegg/

^2^ https://docs.scipy.org/doc/scipy/
