# Supplementary figures and images for: Integrated microbiome-metabolome profiling unveils a predictive signature for early recurrence in hepatocellular carcinoma
Source: Front Microbiol. 2025 Sep 2;16:1653249. doi: 10.3389/fmicb.2025.1653249 (PMC12442831; doi:10.3389/fmicb.2025.1653249)

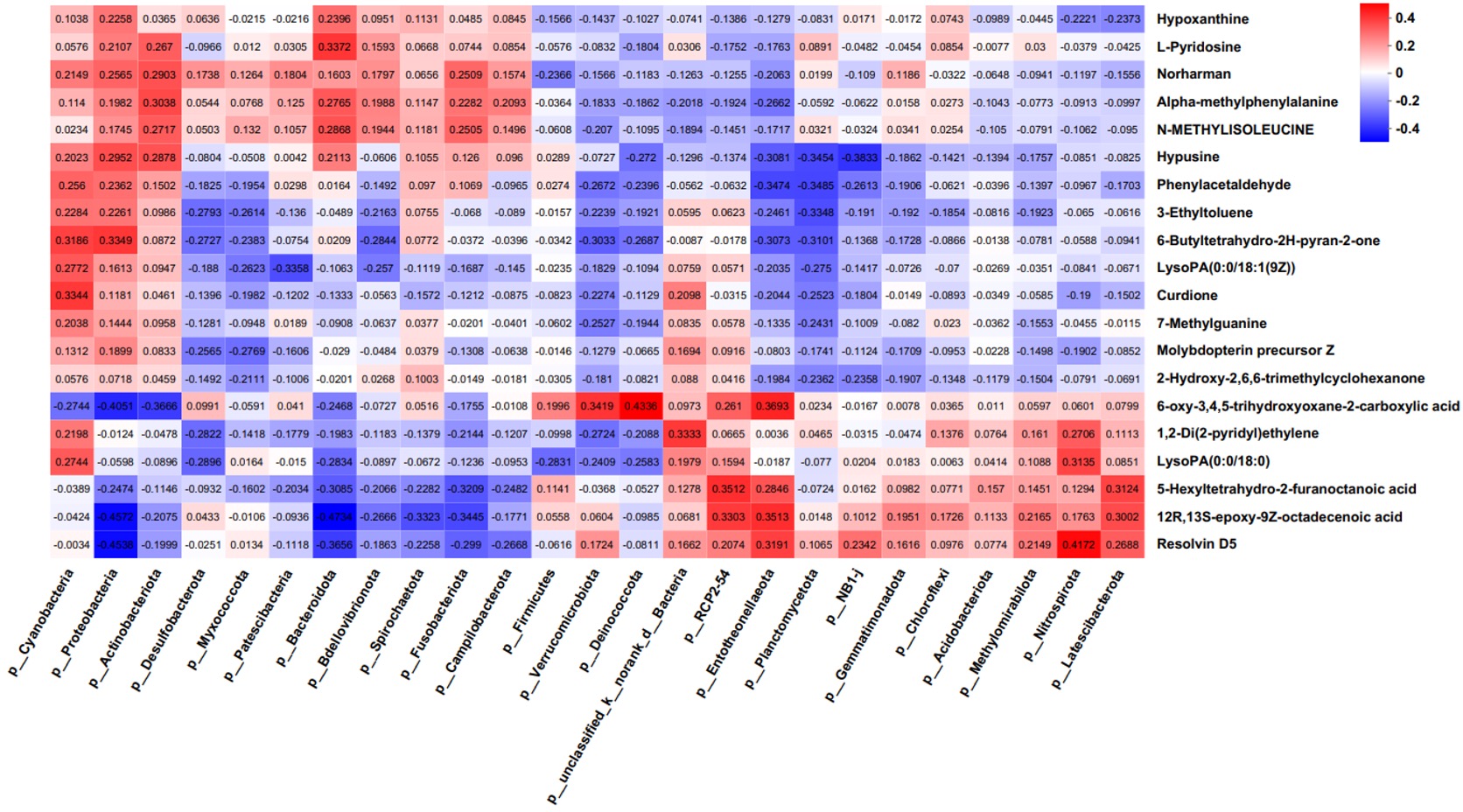

Supplement: Supplementary file 1 [file Image_1.JPEG]
